# Dr. Reiter’s Letter—Lappa Major

**Published:** 1882-11-20

**Authors:** 


### DR. REITER'S LETTER.

### LAPPA MAJOR.

Radix bardanæ, P.G.—Bardane, Fr.—Burdock, E.—Klettenwurzel, G.—Nat. Ord. Compositæ. Cynareæ.

Heads discoid, homogamous; involucre globous, the scales imbrecated and hooked at the extremity; receptacle bristly; pappus bristly, scabrous, caducous, (2) coarse. European herbs. Leaves alternate, large. Lappa Major, Gaert.

Leaves cordate, unarmed, petioled. Common in waste and uncultivated grounds and fields in the New England, Middle and Western States. Each plant is a large, conical, ill-scented and coarse-looking mass of vegetation, surmounted by a branching, irregular panicle of ovoid heads, with tubular corollas of an exceedingly delicate pink color. The leaves are very large, with wavy edges. It has a wonderful design for the dispersion of its seeds. The scales of the involucre all end in a minute, firm hook, which seizes hold of everything that passes by.

The root has long been used as a medicine, particularly in Germany, and alterative, diuretic and diaphoretic properties were ascribed to it. It was prescribed for invalids suffering with rheumatism, skin and other chronic diseases, in powder, infusion, decoction, syrups, etc., but never attained a greater popularity than mint, chamomile, balm and other remedies which the good housewife stores in her medical armamentarium.

It was my misfortune to inherit, from my father, Psoriasis Inveterata, which he told me he had inherited from a long line of progenitors. In youth I had spots on my skin foretelling what adult age developed—psoriasis on left leg and ankle—the same place on my body perfectly imitating my father's plague. He was never healed, although he sought medical advice in Europe.

I was a country doctor, and carried a cane on horseback to relieve the agonizing itching of my leg in warm weather. One warm afternoon (about 1840, I think) I was going to visit a patient with an old farmer, when he exclaimed, "What makes you tear your leg so furiously with the end of that hickory stick?" I replied, "To relieve the maddening itch of my accursed tetter." He said I must cure it; told me he had been afflicted with it on his hands so severely that he had lost his nails. He said I should gather burdock seed and put whisky on it, and take internally. I obeyed; put quite a quantity into gallon bottles and added whisky, of which I had but little; in the others I put alcohol, and stood them in a warm place. After some weeks I began to take a tablespoonful thrice daily, using that steeped in whisky first.

After taking all the whisky tincture I found slight improvement in tetter, but a vast power had been bestowed on my stomach. All my life I had to deny myself many things or suffer; now I could eat sauerkraut, turnips, mince-pie, etc., etc., and only knew I had a stomach from that singular delight we all feel in satisfying a keen appetite with luscious food.

I now began to take alcoholic tincture and found an entirely different preparation. I had to add water and discovered resin, and, at the bottom of each bottle, oil; here was a hint. I put alcohol on the seed which had been macerated in whisky and obtained a resinous tincture. When my old friend and benefactor prepared his tincture, whisky was distilled in copper stills and came off as proof spirits; my whiskey was manufactured by steam. My disease improved rapidly and ceased to torture me, although the skin remained dry and furfuraceous.

On the advent of summer I observed in washing my hands (a doctor must do that often) a great increase in sebaceous secretion, which required much soap to remove; now my whole cutaneous surface acquired a condition of the most perfect health. Whilst afflicted with tetter I had learned to eschew hog meat—a meal of sausages or ham was always very aggravating. I became a very sincere and faithful Jew in avoiding swine meat or fat as a diet. For almost forty years I had a healthy skin; but my European tour in 1875 restored my old malady in an aggravated form. Their water, in many of the southern parts full of dolomite, both for drinking and washing, may have contributed; but sandwiches of cold ham and Bologna sausages were the chief enemies. On my return I could not get the tincture, and, when obtained, its effects were not as before. The taste was not a pure, agreeable bitter, but nauseous. Whether this was owing to mould on the seed or to the druggist having ground them, or to the climate, I could not tell; Mr. Holland said he had bought the seed in New York.

A pupil of mine, Dr. Clark, took my place (in Mount Pleasant, Westmoreland county, Pa.) when I came to Pittsburgh. He was so kind as to have quite a lot of seed gathered for me; I prepared the tincture myself and am now perfectly well. I felt that it restored a perfect digestive power which my stomach lacked, and hence have prescribed it in atonic dyspepsia with such success that Mr. Holland can with difficulty supply the demand: the crop last summer was impaired so much by drought that he was compelled to order the seed from several western States, and still believes he will run short before the new seed can be gathered. I

send to you some of the oil obtained from the bottom of a tincture bottle; some resin I found on a board last fall under a bushel percolator, from which I thought dripping had ended; I send two kinds of tincture, one obtained from contused, the other from whole seed. It can be made in a few days by heating alcohol and keeping warm; the cold preparation is very tedious; much alcohol is lost, the seed absorbing it. We have lost much resin, which clung to percolator and seed: we now save that by washing seed with warm alcohol, which we use for our fresh seed. The therapeutical action I could best reduce to form by calling it an alterative stomachic; it appears to improve all the nutritive, secretive and assimilative functions. I prescribe from two to four drachms, well watered, a half hour before each meal.

I have for a long time wished to send to you something on this Bardanas seed, but am lazy. A suffering illness has admonished me to do so before I die, and I am doing it before I can leave my room. You are the proper person to introduce a remedy and promulgate one of the most valuable therapeutic agents in my armamentarium medicorum.—*Squibs' Ephemeris.*

